# Calculating the absolute binding free energy of the insulin dimer in an explicit solvent

**DOI:** 10.1039/c9ra08284k

**Published:** 2020-01-03

**Authors:** Qiankun Gong, Haomiao Zhang, Haozhe Zhang, Changjun Chen

**Affiliations:** Biomolecular Physics and Modeling Group, School of Physics, Huazhong University of Science and Technology Wuhan 430074 Hubei China cjchen@hust.edu.cn

## Abstract

Insulin is a significant hormone in the regulation of glucose level in the blood. Its monomers bind to each other to form dimers or hexamers through a complex process. To study the binding of the insulin dimer, we first calculate its absolute binding free energy by the steered molecular dynamics method and the confinement method based on a fictitious thermodynamic cycle. After considering some special correction terms, the final calculated binding free energy at 298 K is −8.97 ± 1.41 kcal mol^−1^, which is close to the experimental value of −7.2 ± 0.8 kcal mol^−1^. Furthermore, we discuss the important residue–residue interactions between the insulin monomers, including hydrophobic interactions, π–π interactions and hydrogen bond interactions. The analysis reveals five key residues, Vla^B12^, Tyr^B16^, Phe^B24^, Phe^B25^, and Tyr^B26^, for the dimerization of the insulin. We also perform MM-PBSA calculations for the wild-type dimer and some mutants and study the roles of the key residues by the change of the binding energy of the insulin dimer.

## Abstract 2

In this paper, we calculate the absolute binding free energy of an insulin dimer by steered MD method. The result of −8.97 kcal mol^−1^ is close to the experimental value −7.2 kcal mol^−1^. We also analyze the residue–residue interactions.

## Graphical abstract

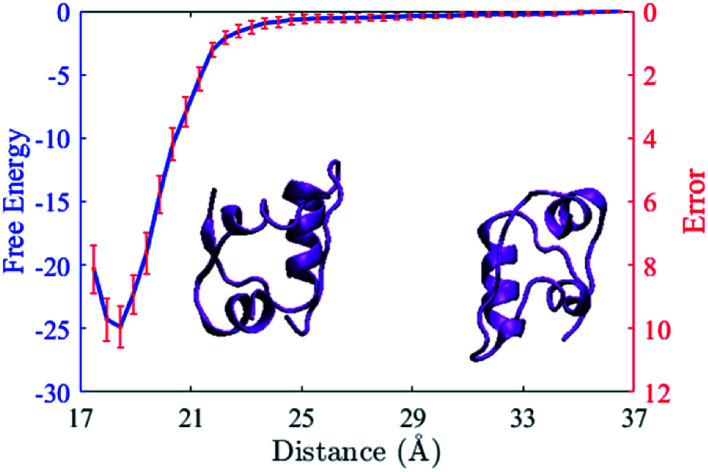

## Introduction

Insulin is a crucial hormone which regulates glucose level in blood. Usually it is stored in the pancreas as a hexamer, and it only shows the biological activity in a monomeric form.^1,2^ The whole process follows three steps in the physiological conditions. First, an insulin hexamer dissociates into dimers and the zinc ions. And then each dimer further dissociates into the monomers. At last the monomer interacts with its receptor.^1^ Nowadays, there remain many challenges in the study of insulin.^2,3^ For example, how to figure out the process by which one insulin monomer binds to another and how to reduce the affinity between them. For the first problem, despite many studies having been done in this field,^4–8^ the real binding mechanism of the insulin monomers is still unclear.^9^ In order to solve the second problem, some fast-acting insulin analogues with low affinities have been exploited, such as lispro insulin (Lys^B28^, Pro^B29^),^10^ and insulin glulisine (Lys^B3^, Glu^B29^).^11^

Determination of the binding affinity of a complex in an experiment is difficult because of time and money. Compared to the experiment, molecular simulation on the computer has an advantage of low cost. It becomes an important assistant tool at present. Based on the molecular simulations, some methods have been developed to predict the affinities of the complexes, such as free energy perturbation molecular dynamics (FEP/MD),^12^ Poisson–Boltzmann surface area (MM-PBSA),^13^ linear interaction energy (LIE),^14^ adaptively biased molecular dynamics (ABMD),^15^ steered molecule dynamics (SMD)^16^ and so on. Among them, SMD may be a promising tool in drug design,^17^ because it is able to produce lots of short and non-equilibrium trajectories.

Now there are two ways to compare the binding affinities of a complex by the SMD method. The first way uses the maximum force (rupture force) on the pulling collective variable to compare the affinities.^18–22^ For example, Mai *et al.* study two ligands binding to influenza virus and find that there is a strong relationship between the maximum force and the experimental free energy. The correlation coefficient is about 0.97.^19^ However, a recent study also indicates that there might be at least two different relationships (correlation coefficients) between the maximum force and the experimental value.^23^ For a specific complex, the real correlation depends on the predominant interaction type between the receptor and the ligand, like the electrostatic interaction, hydrophobic interaction or H-bonds. It makes the comparison work much complicated.

The second way of comparison is calculating the free energy profile.^16^ It is based on an equality proposed by Jarzynski that connects the external work and the free energy differences.^24^ With the equality, one can obtain the free energy profile and the related binding free energy of a complex by lots of non-equilibrium trajectories. The whole calculation is quite suitable for parallelization. Up to now, there are numerous successful applications, like two ligands binding to FKBP protein^25^ and numerous ligands binding to protein FKBP, trypsin and cyclin-dependent kinase 2.^26^ These studies present that the structural flexibility and the steered speed have large impact on the calculations of the free energies. To obtain accurate free energy, the low speed should be used in SMD simulation. Moreover, to improve the calculation accuracy, a lot of practical methods have been proposed, including the fluctuation-dissipation theorem of Brownian dynamics (BD-FDT),^27^ adaptive SMD,^28^ hybrid SMD,^29,30^ and the non-equilibrium friction correction method.^31^

A few years ago, Tyka and co-workers proposed a confinement method to calculate the free energy differences between two different stable states of a single molecule.^32^ Suppose these two stable states are P and Q, respectively. The free energy difference between the two states is Δ*F*_PQ_. Based on a thermodynamic cycle, the calculation of Δ*F*_PQ_ is divided into three independent stages. Two of them are the calculations of the free energy differences, Δ*F*_PP*_ and Δ*F*_QQ*_. They correspond to the transitions from the flexible state, P and Q, to the restrained state, P* and Q*, respectively (method: thermodynamic integration (TI)^33^). The last stage is the calculation of the free energy difference between the two restrained states P* and Q* (method: normal mode analysis (NMA)^34^). The confinement method has been shown to be practical for an isolated molecule. Recently, Perthold and Oostenbrink also construct a thermodynamic cycle to calculate the binding free energies of some protein–protein complexes with the perturbed distance restraints.^35^ Their free energy results are close to the experimental values.

In this work, we borrow the ideas of the above papers into the calculation of the absolute binding free energy of an insulin dimer system. Since TI and NMA are very time-consuming for large systems, we replace them by SMD. In our work, one SMD simulation is performed in a virtual binding process of the two insulin monomers. To remove the effect of the restraints from the results, we also perform two additional SMD simulations to produce the free energy differences between the restrained and unrestrained states. Just as the work in ref. 35, the three processes constitute a fictitious but complete thermodynamic cycle from the unbound state to the bound state for the insulin monomers. The final result in our work is −8.97 ± 1.41 kcal mol^−1^, which is close to the experimental value −7.2 ± 0.8 kcal mol^−1^.^36^

Besides the calculation of the absolute binding energy by SMD, we also investigate the binding mechanisms of the insulin dimer. A set of simulations are performed for the dimer at different distances. The results show the importance of the π–π interaction, hydrophobic interaction and the hydrogen bond interaction in the dimerization process. Finally, we perform the MM-PBSA calculations^13^ to study how the key residues affect the binding energies of the dimer.

## Materials and methods

### Structural information

**Fig. 1:**
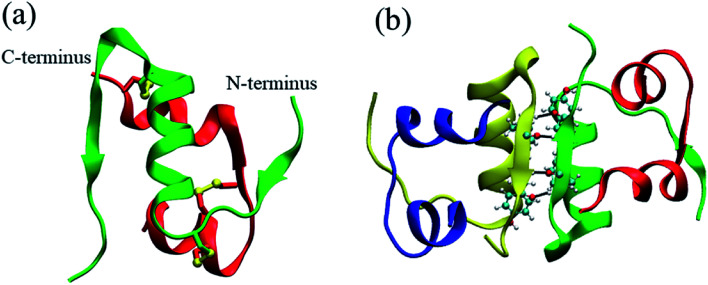
(a) Monomeric structure from the pig insulin dimer in Protein Data Bank^53^ (PDB ID: 4INS^54^). Chain A and chain B are rendered in red color and green color, respectively. Disulfide bonds, Cys^A20^–Cys^B19^, Cys^A6^–Cys^A11^, Cys^A7^–Cys^B7^ from top to bottom, are plotted in the yellow balls and sticks model. (b) Structure of the pig insulin dimer from the Protein Data Bank^53^ (PDB ID: 4INS^54^). Four hydrogen bonds between the two monomers are represented by the black solid lines. The figure is produced by VMD.^55^

The conformation of the insulin dimer is shown in Fig. 1(b). Its monomeric structure consists of two chains (Fig. 1(a)). Chain A in red color has 21 residues and chain B in green color has 30 residues. They are associated with each other by two disulfide bonds (Cys^A7^–Cys^B7^, Cys^A20^–Cys^B19^). Two monomers bind together to form a dimer (Fig. 1(b)). On the interaction surface between the monomers, there is a hydrophobic core including two antiparallel β-sheets (B24–B26) and two α-helixes (B9–B18). And the β-sheets are stabilized by four hydrogen bonds (Fig. 1(b)). Many experiments have been done to investigate the structure and dynamics of insulin, such as atomic force microscopy (AFM) experiment,^37^ alanine scanning experiment,^38^ amide I two-dimensional infrared spectroscopy (2D IR) experiment,^39^ laser induced temperature jump (T-jump) experiment,^40^ time-resolved X-ray scattering experiment^41^ and so on. Moreover, at 298 K, concentration difference spectroscopy experiment presents that the insulin dimer's binding free energy is −7.2 ± 0.8 kcal mol^−1^ (ref. 36) and the isothermal titration microcalorimetry (ITC) dilution experiment gives its dissociation free energy 6.88 ± 0.03 kcal mol^−1^.^42^ These studies suggest that both of the insulin dimerization and dissociation are enthalpy control. As to the simulations, the dynamic properties of the insulin monomer,^43,44^ dimer^45–50^ and hexamer^51,52^ in water are also investigated by some groups. For instance, two distinct surfaces (the dimer forming surface and the hexamer forming surface) on an insulin monomer are characterized in ref. 44. The role of the confined water molecules to the stability of the insulin hexamer is studied in ref. 52.

### Thermodynamic cycle

Since insulin tends to exist as dimers or hexamers in aqueous solvent, the monomeric structure of the insulin is still unknown in experiment. In an early NMR experimental study, the monomeric structure of an insulin variant des-[Phe^B25^] (insulin without the Phe^B25^ residue) was determined.^56^ It does not show a large structural difference to that in the dimer of the wild insulin. To verify the structural stability, we extract the monomer from the crystal structure of the insulin dimer and perform four independent 100 ns equilibrium simulations. The fluctuations of the RMSD of C_α_ atoms are about 2.0 Å (the first two and the last two residues are not included). It proves again that the monomeric structure from the dimer is relatively stable in aqueous solvent.

**Fig. 2:**
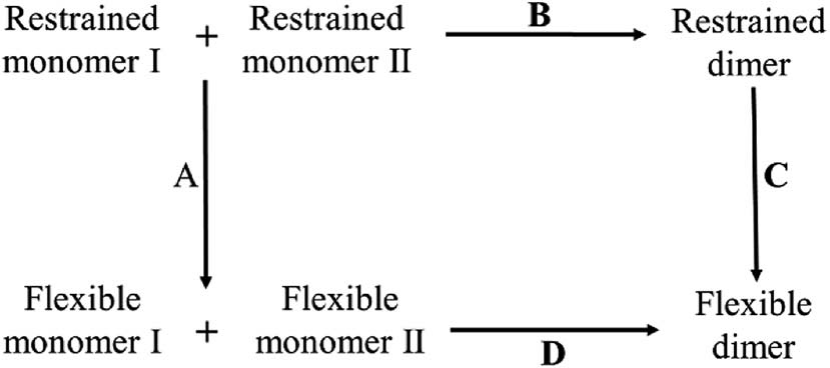
Thermodynamic cycle in the calculation of the absolute binding free energy of insulin dimer. Process D at bottom is the real binding process of a dimer. All the molecules at the bottom are flexible in the process. Process A, B and C are the virtual processes for SMD simulation.

In 1985, concentration difference spectroscopy experiment obtained the thermodynamic quantities of porcine insulin, including the changes of enthalpy, entropy and free energy in the binding process.^36^ The experimental values can be used as reference but the process is too complicated to be simulated on computer, so does the calculation of the absolute binding free energy. In current work, inspired by the confinement method^32^ and the work by Perthold *et al.*,^35^ we also use a thermodynamic cycle to circumvent the issue. The schematic diagram of the cycle is illustrated in Fig. 2.

In the figure, the whole thermodynamic cycle consists of four independent processes. Process D at bottom is corresponding to the real binding process from the unbound flexible monomers to the dimer. Process A, B and C are the virtual processes to close the thermodynamic cycle. Among them, process A at left is a virtual relaxing process from the restrained unbound state to the flexible unbound state. In the restrained unbound state, eleven geometric restraints are applied to the system (see the details below). And in the flexible unbound state, all the restraints are all removed from the system. Process C at right is similar to process A except that the SMD simulation is only performed for the bound state. As to process B at top, it is a virtual binding process from the unbound state to the bound state with a fixed force constant in all the restraint potentials. It must be noted that the simulation of the virtual binding process B is much easier than the real binding process D. This is due to the decrease of the degrees of freedom in the system.

Based on the thermodynamic cycle, we divide the calculation of the absolute binding free energy of insulin dimer Δ*F*_D_ into three parts, Δ*F*_A_, Δ*F*_B_ and Δ*F*_C_. Each part represents a free energy difference in the corresponding process. All the simulations are performed by SMD.^16^ Finally, the absolute binding free energy of insulin dimer is obtained by the following formula:

1 Δ*F*_D_ = −Δ*F*_A_ + Δ*F*_B_ + Δ*F*_C_

### Standard-state correction and rotational restriction correction

In this work, we also involve the standard-state correction Δ*F*_V_ and the rotation restriction correction Δ*F*_R_. Δ*F*_V_ gives the free-energy difference when one molecule transfers from the available volume in the unbound state *V*_u_ to the standard-state volume *V*_0_ (1661 Å^3^).^35,57^2
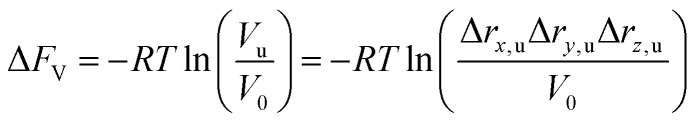
Here Δ*r*_*x*,u_, Δ*r*_*y*,u_ and Δ*r*_*z*,u_ are the differences between the minimum and maximum monomer–monomer distances in the *x*, *y* and *z* directions at the unbound state with restraints, respectively.

The rotational restriction correction term Δ*F*_R_ is introduced to account for the effect of the restraints in the bound state, which is given in the following formula:^35^3
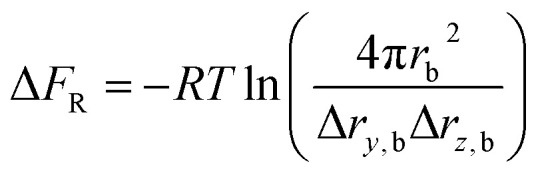
Here Δ*r*_*y*,b_ and Δ*r*_*z*,b_ are the maximum deviations of the distances in the *y* and *z* directions at the bound state with restraints, and *r*_b_ is the average radial monomer–monomer distance in the bound state without restraints. As a consequence, the absolute binding free energy of the insulin dimer becomes

4 Δ*F* = −Δ*F*_A_ + Δ*F*_B_ + Δ*F*_C_ + Δ*F*_V_ + Δ*F*_R_

We want to note that the reaction coordinate in the SMD simulation for the virtual binding process B is the distance between the monomers along a fixed *X*-axis. The free energy does not need the Jacobian correction.^58^

### Free energy calculation

Here we give a brief introduction of the existing SMD method. Thermodynamics theory says that when a system changes infinitely slow along a path from its state X to state Y, the Helmholtz free energy difference between the states equals the external work *W* performed on this system:

5 *W* = Δ*F* = *F*_Y_ − *F*_X_

where *F*_X_, *F*_Y_ is the free energy of state X and state Y, respectively. This equation only works for an extremely slow process. As to a transition process with a finite rate, Jarzynski proposes a new equality:^24^

6 exp(−*β*Δ*F*) = 〈exp(−*βW*)〉

Here *β* is the inverse temperature 1/*k*_B_*T*, and the bracket indicates an ensemble average. This equality allows us to obtain the free energy difference from lots of non-equilibrium short trajectories.

To force the system to move along a fast transition path, some kind of restraint potential should be applied. The potential may have many different forms,^59^ such as that with the constant forces or torques. But the most widely used one is the spring potential. It has the following form:7
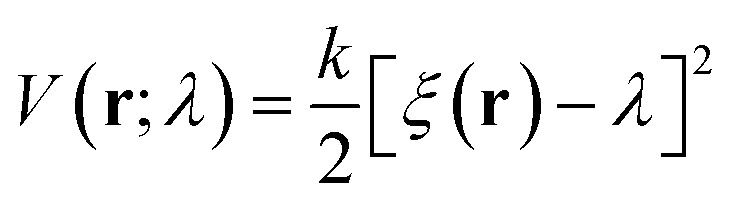
where **r** is the positions of the atoms in a 3N dimensional space. *ξ*(**r**) is the collective variable to be restrained on the path. In this work, it is set as the distance of center-of-mass of the insulin monomers at *X*-axis for process A, B and C. *k* is the force constant of the restraint potential. The center of the spring potential, *λ*, moves according to following formula:

8 *λ* = *λ*_0_ + *vt*

*λ*_0_ is the initial position of the system on the path, *t* is the simulation time and *v* is the constant moving velocity.

With such spring potential, the new Hamiltonian of the restrained system is

9 *H*(**r**,**p**;*λ*) = *H*_0_(**r**,**p**) + *V*(**r**;*λ*).

Here *H*_0_ is the Hamiltonian of the original system and **p** is the momenta of the atoms. According to the Jarzynski's equality, the free energy on the path is given as follows:

10 exp{−*β*[*F*(*λ*_*t*_) − *F*(*λ*_0_)]} = 〈exp(−*βW*_0→*t*_)〉

*W*_0→*t*_ is the external work done on the system when it moves from *λ*_0_ to *λ*_*t*_ on the path. The work can be calculated by an integral on time from 0 to *t*:11
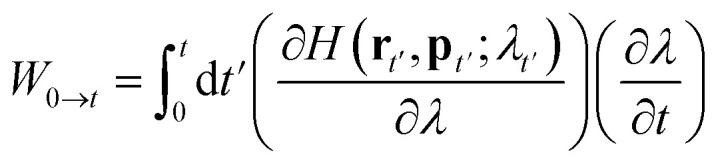


Substitute the restrained Hamiltonian into the formula, the external work becomes:12
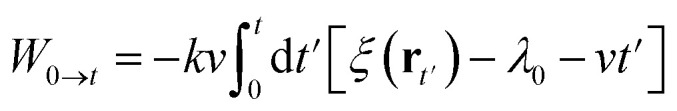


### Definition of the restraint potentials

Besides the distance of center-of-mass, there are ten more restraints on the two insulin monomers. These restraints are used to ensure the monomers to form a correct complex conformation in the SMD simulation. The first two restraints are the heavy atoms RMSD restraints. They restrain the structures of the individual monomers in the virtual binding process. The third and fourth restraints are the center-of-mass (C_α_ atoms) restraint for each monomer, which is used to ensure the monomers move along the *X*-axis direction (binding direction). The restraint potential has the form13
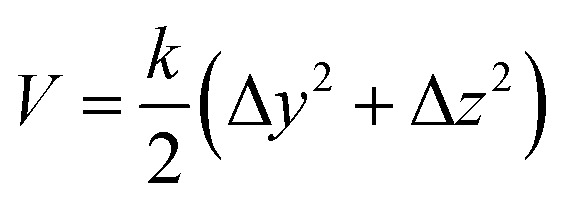


Here Δ*y* and Δ*z* are the deviations of the center-of-mass of the monomers to the *X*-axis in the *y* or *z* direction, respectively. The form of the restraint potential does not change with time. And it does not have the steered coordinate in the SMD calculation (center-of-mass distance between the monomers on the *X*-axis).

**Fig. 3:**
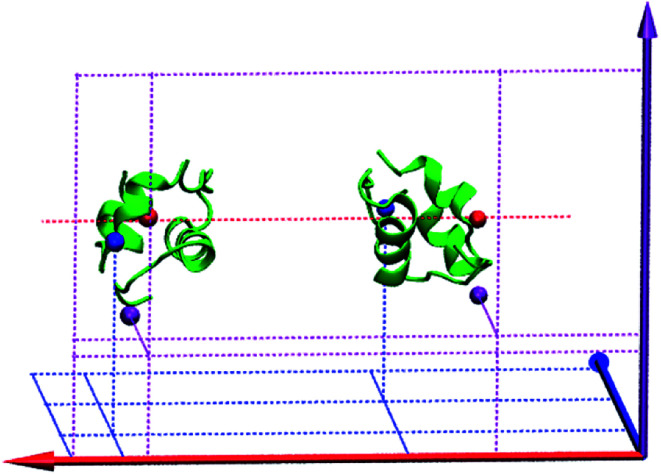
Six single-atom restraints in the SMD simulation for the insulin dimer. The red, purple and blue solid arrows represent the *X*, *Y* and *Z* axis, respectively. Two red atoms are fixed at the horizontal red dotted line, two purple atoms are fixed on the vertical *X*–*Y* plane with a fixed *z* coordinate and two blue atoms are fixed on the horizontal *X*–*Z* plane with a fixed y coordinate. The figure is produced by VMD.^55^

The rest are six restraints on six different atoms of the monomers. They are used to avoid the rotation of the monomers. As shown in Fig. 3, two red atoms (OE1 of Gln^A15^ in monomer I and CD1 of Leu^A16^ in monomer II) are fixed on the red horizontal dotted line. Two purple atoms (ND1 of Hid^B5^ in monomer I and CG1 of Val^B2^ in monomer II) are fixed on the purple vertical *X*–*Y* plane with a fixed *z* coordinate. Two blue atoms (N of Lys^B29^ in monomer I and CA of Thr^A8^ in monomer II) are fixed on the blue horizontal *X*–*Z* plane with a fixed *y* coordinate. All these restraints are the harmonic potentials with a force constant of 50 kcal mol^−1^ Å^−2^.

### Simulation details

The initial structure of insulin dimer comes from the Protein Data Bank^53^ (PDB ID: 4INS^54^). First, the complex is rotated to be aligned with *X*-axis (red solid arrow in Fig. 3). Then it is solvated in a periodic box of 88.6 Å × 55.5 Å × 55.3 Å with 6498 TIP3P water molecules.^60^ Na^+^ and Cl^−^ ion concentration is 12 mM, which is similar to experiments.^36^ Second, the solvated system is minimized for 10 000 steps by the steepest descent method and 10 000 steps by the conjugate gradient method. Third, a 20 ns equilibrium simulation is performed to relax the system. In the simulation, the center-of-mass of each monomer is restrained to *X*-axis. Finally, the distance between the monomers is gradually increased to 36.5 Å with the restraints mentioned above. The final unbound conformation of the system is shown in Fig. 3.

In this work, the force field is ff14SB AMBER force field^61^ and the time step is 2.0 fs. SHAKE algorithm^62^ is used to constrain the bonds involving the hydrogens. The non-bonded interaction is calculated with a cut-off of 10.0 Å. All the SMD simulations are performed by AMBER 18 (ref. 63) in the NVT ensemble at 298 K.

In the virtual binding process B in Fig. 2, the center-of-mass distance between the insulin monomers decreases from 36.5 Å to 17.5 Å with a low speed of 0.2 Å ns^−1^. All the restraints are kept in the simulation. The simulation time of the process lasts for 95 ns. Process A and C are also simulated by SMD but with a little difference. In these two processes, the driven coordinate is the force constant *k* in the restraint potential that changes from a restrained state (*k* = 50 kcal mol^−1^ Å^−2^) to a flexible state (*k* = 0 kcal mol^−1^ Å^−2^). As a comparison, a standard SMD uses the distance as the driven coordinate. The simulation time of process B and C is 20 ns. To obtain the average free energy difference, we perform 40 independent simulations for every process.

To study the residue–residue interactions in the dimerization of the insulin (hydrophobic interaction, π–π interaction and hydrogen bond interaction), we run five simulations at a center-of-mass distance of 26 Å, 24 Å, 22 Å, 20 Å and 18 Å (native state). Each simulation consists of eight independent 100 ns trajectories, whose initial structure is extracted from process B and further equilibrated for 20 ns. To fix the distance between the monomers, an umbrella restraint potential *U* is applied to the system in the simulation.14
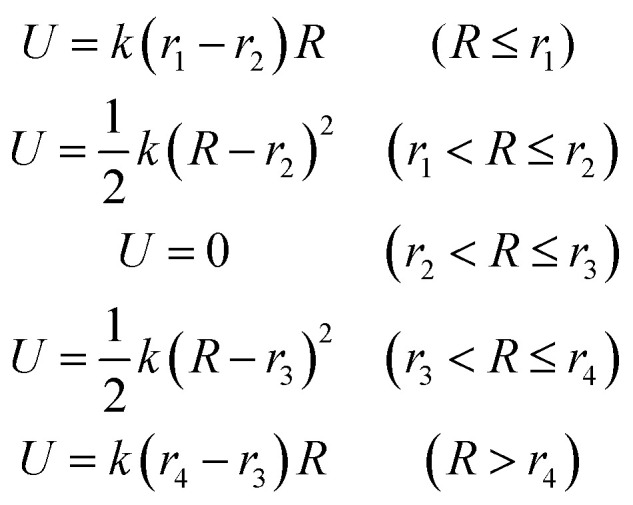
Here *R* is the center-of-mass distance between the monomers. *k* is 50 kcal mol^−1^ Å^−2^. *r*_1_, *r*_2_, *r*_3_ and *r*_4_ determine the region. For instance, in the simulation at a distance of 26.0 Å, *r*_1_, *r*_2_, *r*_3_ and *r*_4_ are set to 25.5 Å, 25.6 Å, 26.4 Å and 26.5 Å, respectively.

We also perform MM-PBSA calculations to get the change of the binding energy between the wild-type dimer and the mutants. To get the mutants, we remove all the sidechain atoms of the selected residues except the C_β_ atom. And then change their names to ‘ALA’ in the pdb file. The missing hydrogen atoms are added automatically by the tleap tool in AMBER 18.^63^ In all the MM-PBSA calculations, we use the three-trajectory mode and run 27 trajectories for each mutant. For each trajectory, the simulation time is 100 ns and 25 snapshots are extracted evenly from the last 50 ns. So, in total we have 225 snapshots in the MM-PBSA calculation. The calculation is performed by the MMPBSA.py script in AMBER 18.^63^ The dielectric constants of the solute and the solvent are 1.0 and 80.0, respectively. The nonpolar part of the solvation energy is estimated by:

15 **G**_np,solv_ = 0.0072 × SASA

## Results and discussions

As shown in the thermodynamics cycle in Fig. 2, the calculation of the binding free energy of the insulin dimer is completed by three independent processes. The main process is the virtual binding process from the restrained unbound state to the restrained bound state (process B). And the other two are the virtual relaxing processes at the unbound and bound state, respectively (process A and C). For each process, we perform a repeated SMD simulation^16^ to generate 40 trajectories. These trajectories are used for the calculation of the free energies (eqn (10)) and the corresponding errors.

**Fig. 4:**
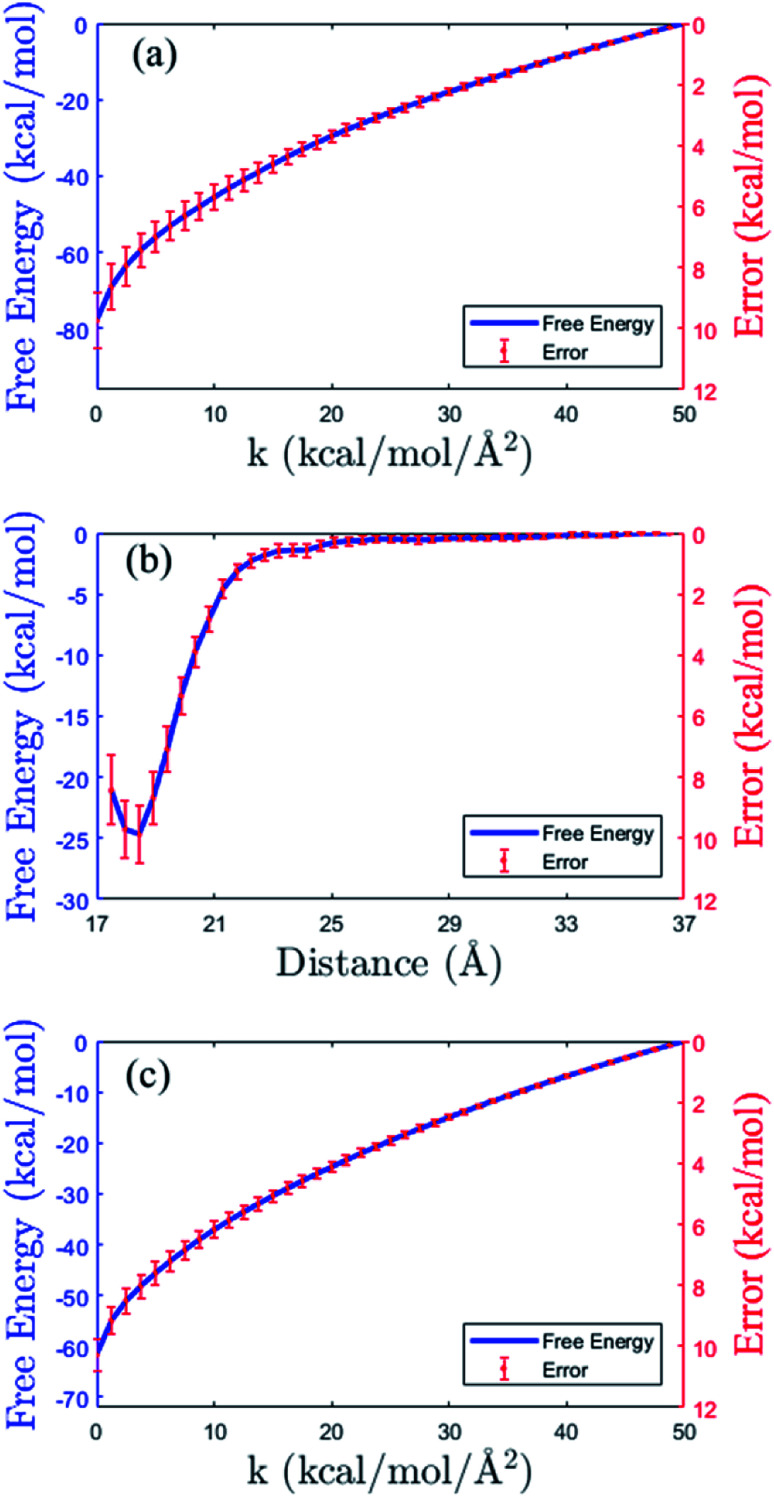
Free energy profiles (blue solid lines) and the errors (in red color) in the three processes of the thermodynamic cycle in Fig. 2. (a) Virtual relaxing process A. (b) Virtual binding process B. (c) Virtual relaxing process C. See the descriptions of the processes in the main text.

The free energy profiles of the three processes are shown in Fig. 4. Panel (b) is the data of the virtual binding process B. In the process, the center-of-mass distance between the monomers decreases from 36.5 Å to 17.5 Å. And the least free energy Δ*F*_B_ is −25.02 ± 0.95 kcal mol^−1^ at distance 18.2 Å. Panel (a) and (c) are the data of the two virtual relaxing processes A and C at the unbound state (distance 36.5 Å) and bound state (distance 18.2 Å), respectively. In these two processes, the force constants in all the restraint potentials decrease from 50.0 kcal mol^−1^ to 0 kcal mol^−1^ gradually. So, at the end of the processes, the molecule turns into a flexible structure completely. The end-to-end free energy differences of the two processes are −77.88 ± 0.89 kcal mol^−1^ and −61.66 ± 0.53 kcal mol^−1^, respectively. As expecting, all the free energy errors increase when the molecule moves along the path.

In order to have the absolute binding free energy, the standard-state correction and the rotational restriction correction are both considered in our work. The standard-state correction (eqn (2)) is 5.45 kcal mol^−1^ and the rotational restriction correction (eqn (3)) is −5.62 kcal mol^−1^. Combining all these data together, the final absolute binding free energy of the insulin dimer is −8.97 ± 1.41 kcal mol^−1^ (eqn (4)). In experimental aspect, the pig insulin dimer has been studied by the concentration difference spectroscopy. Strazza *et al.* measure the equilibrium constant *K*_a_ at five uniformly-spaced temperatures from 394 K to 311 K.^36^ At 298 K, the corresponding binding free energy is −7.2 ± 0.8 kcal mol^−1^.^36^ It shows that our calculation result is close to the experimental data.

In a previous study, Zoete *et al.* calculate the absolute binding free energy of the insulin dimer by the MM-GBSA method.^46^ In this method, the binding free energy is constituted of an enthalpy term and an entropy term. The former includes the difference of the gas phase energy and the solvation free energy between the unbound and the bound state. And the latter contains the difference of the transitional, rotational and vibrational entropies. The MM-GBSA result is −11.91 ± 6.7 kcal mol^−1^,^46^ which is comparable to the experimental value.^36^ But, its enthalpy term and the entropy term are −38.65 ± 5.8 kcal mol^−1^ and −26.74 ± 3.6 kcal mol^−1^, respectively.^46^ The experimental enthalpy and entropy (*T*Δ*S*) is −10.0 kcal mol^−1^ and −3.3 kcal mol^−1^ respectively, which are obtained approximately by van't Hoff relation. These two large quantities are greatly different to that in experiment. This may be because the MM-GBSA method includes many parameters and approximations.^13,34,64,65^ For the enthalpy term, the error might come from the implicit solvent model,^13,66^ and for the entropy term, the error might come from the harmonic approximation.^34^ And there are some improved methods aim to decrease the errors in the MM-GBSA or MM-PBSA calculation.^67–70^

Compared to MM-GBSA, SMD is quite simple. Its calculation is based on work *W* in eqn (6), instead of the separated enthalpy and entropy terms. But SMD do have its own parameters, such as the steered speed and the number of the trajectories. Previous studies have shown that both slowing down the steered speed and increasing the number of the trajectories can improve the accuracies of the results.^25–27^ However, preparation of a proper restraint is still difficult in SMD.

One more thing we want to note is that SMD completes the calculation in the explicit solvent. And MM-GBSA or MM-PBSA are not. In MM-GBSA or MM-PBSA, the snapshots are extracted from the explicit solvent, but the free energy calculation is done in implicit solvent. This may bring the underlying problem to the calculation of the absolute binding free energy. For example, determination of the reference solute dielectric constant value is not easy. It can be 1.0, 2.0 or 4.0 in different MM-PBSA calculations.^71–74^

**Fig. 5:**
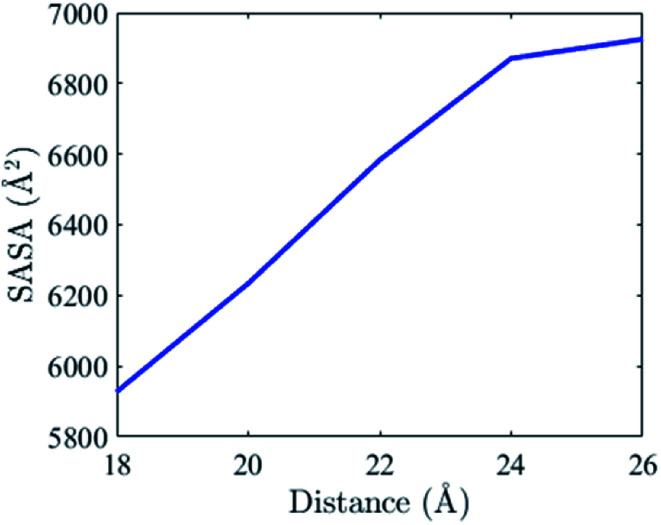
The average SASA of the insulin dimer at different monomer–monomer distance.

Insulin is an essential protein in treating the disease. Understanding the binding mechanism of insulin dimer will be useful to design drug. Fig. 5 shows the change of the average solvent-accessible-surface area (SASA) in the binding process of the insulin monomers. The data come from five independent MD simulations at the center-of-mass (COM) distance of 26 Å, 24 Å, 22 Å, 20 Å and 18 Å (native state), respectively.

**Fig. 6:**
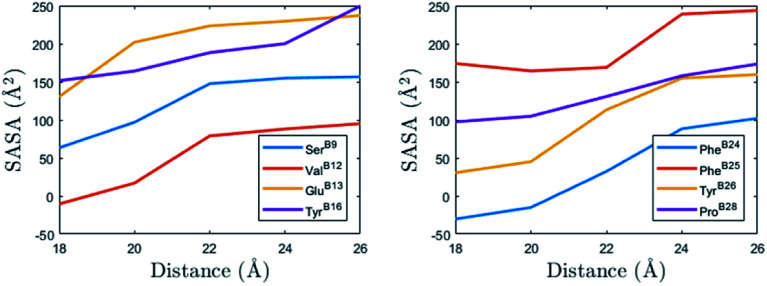
The changes of the SASA of the eight residues in Table 1 from the isolated monomers (distance 26 Å) to the complex (distance 18 Å).

The change of the total SASA in the binding process (COM distance from 26 Å to 18 Å) mainly comes from 22 residues on the binding interface (B9–B30). We present eight residues that have the largest SASA differences in Table 1. Their SASA changes are plotted in Fig. 6. Among these residues, three are hydrophobic residues (Val^B12^, Phe^B24^, Phe^B25^), four are polar residues (Ser^B9^, Tyr^B16^, Tyr^B26^, Pro^B28^) and one is the charged residues (Glu^B13^). According to the definition of the hydrophobic interaction, the SASA reductions of the three hydrophobic residues (Val^B12^, Phe^B24^, Phe^B25^) should favor the binding of the monomers. These residues are important to the insulin dimerization. Moreover, Phe^B24^ and Phe^B25^ are on the flexible β-strand, their hydrophobic interactions also favor the formation of the β-sheet between the monomers.

**Table 1:** Eight residues that have the largest SASA differences from the isolated monomers (distance 26 Å) to the complex (distance 18 Å)

Residue	Phe^B24^	Tyr^B26^	Glu^B13^	Val^B12^	Tyr^B16^	Ser^B9^	Pro^B28^	Phe^B25^
SASA	132.3	129.0	106.7	105.5	98.0	93.2	76.0	69.6

π–π interaction between the aromatic amino acids is a typical non-covalent intermolecular force. Recently, Zhao *et al.* investigate lots of pairs of aromatic amino acids and find some statistical laws.^75^ In their definition, π–π interaction satisfies some geometry conditions. First, the center-of-mass distance between two aromatic rings should be less than 7.2 Å. Second, the normal–normal acute angle should be larger than 50° (T-shaped conformation) or less than 30° (parallel conformation). In this work, we use the definition to study the π–π interactions between insulin monomers.

**Fig. 7:**
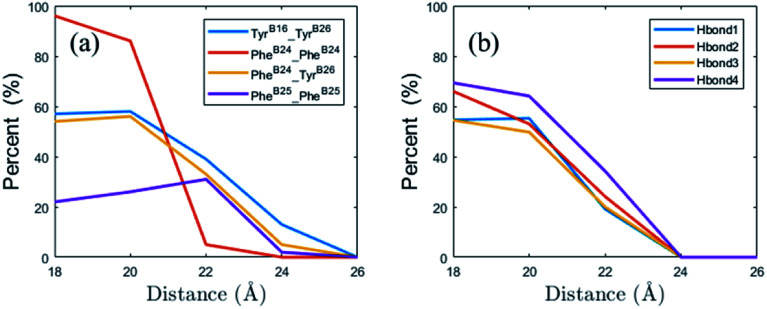
(a) The percent of the four strongest π–π interaction between the aromatic amino acid pairs at different distances between the insulin monomers. (b) The percent of four inter-strand hydrogen bonds at different distances. (O–N distance smaller than 3.0 Å and donor–H–acceptor angle larger than 135°).

There are four aromatic amino acids (Tyr^B16^, Phe^B24^, Phe^B25^ and Tyr^B26^) for each monomer on the binding interface. In the binding process, the percent of the π–π interactions between four strongest aromatic pairs are shown in Fig. 7(a) (Tyr^B16^_Tyr^B26^, Phe^B24^_Phe^B24^, Phe^B24^_Tyr^B26^ and Phe^B25^_Phe^B25^). From the figure, we find that the first three π–π interactions increase quickly in the binding process. Besides the hydrophobic interaction, the inter-strand hydrogen bond interaction is also important to the stabilization of the complex. Fig. 7(b) gives the change of the percent of four inter-strand hydrogen bonds at different distances between the monomers. The locations of these bonds in the complex are shown in Fig. 1. It presents that all the four inter-strand hydrogen bonds become stable at 20 Å (percent larger than 50%). They make the two flexible β-strands form into one global β-sheet.

**Fig. 8:**
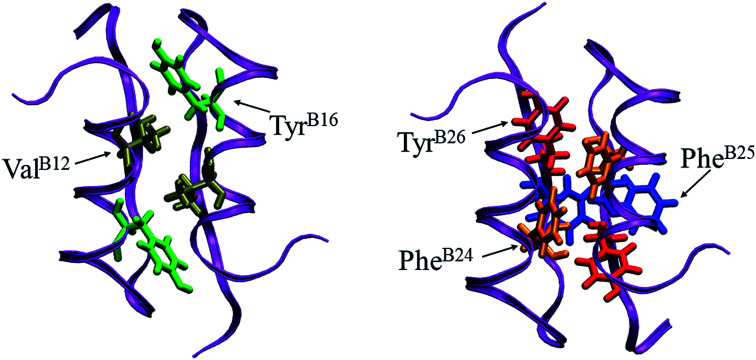
The positions of five key residues on the binding interface of the insulin dimer. They are Val^B12^ (tan), Tyr^B16^ (green), Phe^B24^ (orange), Phe^B25^ (blue) and Tyr^B26^ (red). The figure is produced by VMD.^55^

In a word, the dimerization of the insulin is favored by the hydrophobic interaction, π–π interaction and hydrogen bond interaction. Among the 22 residues on the binding interface, five key residues Val^B12^, Tyr^B16^, Phe^B24^, Phe^B25^, and Tyr^B26^ are picked out according their involvement in the interactions. The locations of the key residues in the dimer are shown in Fig. 8. These findings are close to previous alanine scanning experiment.^38^ The experiment studies some mutants of the insulin dimer (Val^B12^Ala, Tyr^B16^Ala, Phe^B24^Ala, Tyr^B26^Ala, Asn^A21^Ala, Phe^B25^Ala, Thr^B27^Ala) and finds that the first four mutants exist as a monomeric insulin after the mutation. Why these residues (Val^B12^, Tyr^B16^, Phe^B24^, Tyr^B26^) are important to the dimerization of the insulin can be well explained by the analysis of the residue–residue interactions here. Of course, the hydrophobic residue Phe^B25^ is an exception. In the mutation experiment, Phe^B25^ does not present to be critical to the insulin dimerization apparently. In the simulation, its SASA reduction is smaller than the other hydrophobic residues (Table 1). And its π–π interaction is weaker than the other aromatic pairs (Fig. 7(a), magenta line). So, compared to the other key residues, Phe^B25^ may have less contribution to the binding of the monomers.

MM-GBSA or MM-PBSA are the popular approaches to study the relative binding affinity between similar complexes.^76,77^ Because in this case, the entropy contribution can be safely ignored. For instance, Huiyong *et al.* study lots of protein–ligand systems and find that there is a good correlation between the enthalpy value and the binding affinity in experiment.^76^ To verify the idea, we further investigate the variation of the enthalpy of the insulin dimer and its mutants by the MM-PBSA method. Seven residues Asn^A21^, Val^B12^, Tyr^B16^, Phe^B24^, Phe^B25^, Tyr^B26^ and Thr^B27^ are mutated to Ala one by one. The entropy term is not included in the calculation.

Table 2 gives the enthalpy of the wild-type dimer and the mutants. The changes of the structural RMSD in the simulations are also presented. Compared to the wild-type dimer, the three mutants, Val^B12^, Tyr^B16^Ala, Tyr^B26^Ala, have a much higher enthalpy. Mutations on these residues decrease the binding affinities of the dimer remarkably. The results are close to the findings in previous alanine scanning experiment^38^ except the mutant Phe^B24^Ala. It presents that MM-PBSA is successful in the prediction of the relative binding affinity of the complex. As to the average structural C_α_ RMSD, all the mutants show larger RMSD values than the wild-type dimer. The mutations destabilize the structure of the native state.

**Table 2:** The Enthalpy and the structural RMSD of the wild-type insulin dimer and the analogues

Mutant	Enthalpy (kcal mol^−1^)	Dimer RMSD (Å)
Wile-type	−61.4	1.8
Phe^B24^Ala	−62.0	2.2
Phe^B25^Ala	−54.8	2.0
Asn^A21^Ala	−48.7	1.9
Thr^B27^Ala	−48.7	1.9
Val^B12^Ala	−44.4	2.0
Tyr^B26^Ala	−37.7	2.2
Tyr^B16^Ala	−35.7	2.3

**Fig. 9:**
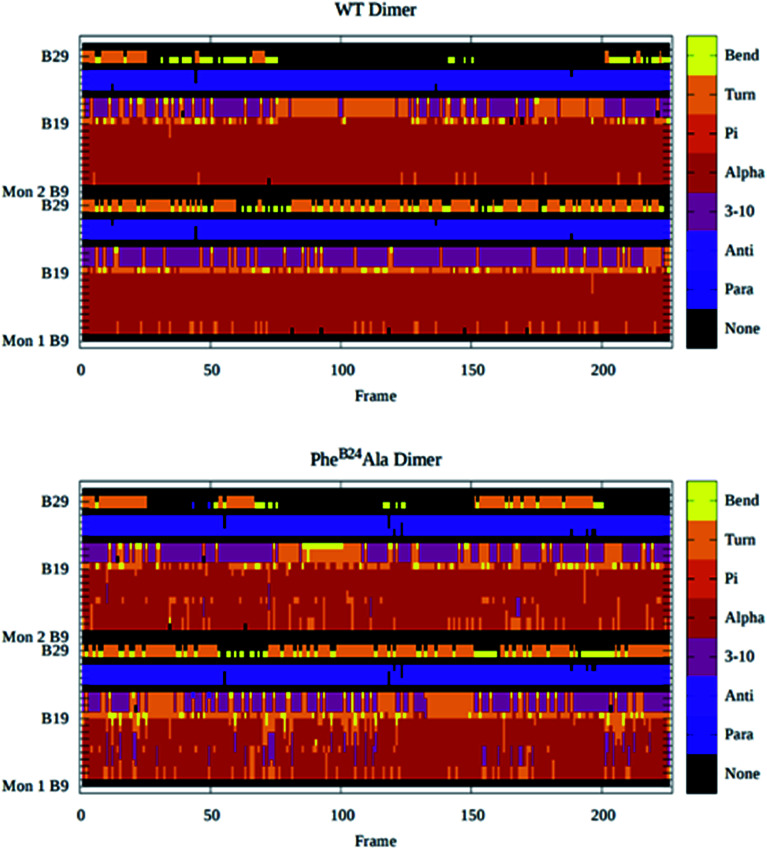
The change of the secondary structures of the wild-type dimer (WT dimer) and the mutant dimer Phe^B24^Ala in the simulation. Different secondary structures are plotted in different colors (defined in the color bar at right). The calculation is performed by DSSP.^78^

To describe the effect of the mutation, we also calculate the secondary structures of the residues on the binding interface (B9–B30) for the wild-type dimer and all the mutants by DSSP.^78^ Among them, the mutant Phe^B24^Ala shows the most difference than the wild-type dimer. The changes of the secondary structures of the wild-type dimer and the mutant Phe^B24^Ala are shown in Fig. 9. In the figure, red color represents the α-helix and purple is the β-sheet. We find that both of the α-helix (B9–B18) and the β-sheet (B24–B26) are quite stable in the wild-type dimer. The dimerization is helpful to the stabilization of the secondary structures. But for the mutant Phe^B24^Ala, the α-helix changes into the “turn” conformation frequently in the whole simulation time. The average percent of the α-helix (B9–B18) decrease from 83.0% (wild-type) to 72.8% (mutant). As to the individual residues in the helix, in monomer I, the percent of Ala^B14^ and Val^B18^ decrease 26.0% and 21.0%, respectively. The other residues, Val^B12^, Glu^B13^, Leu^B15^, Tyr^B16^ and Leu^B17^, decrease about 15%. And in monomer II, the residue Ala^B14^ decreases 19.0%, and Val^B12^, Glu^B13^ and Val^B18^ decrease about 5%.

## Conclusion

Protein–protein association is an important process in cell biology. Many typical systems have been successfully investigated in the simulations, such as the melittin aggregation^79,80^ and the fibritin foldon domain assembly.^81^ In this work, we study the dimerization of insulin. Combing the SMD method^16^ with the confinement method,^32^ we calculate its absolute binding free energy based on a fictitious thermodynamic cycle. The calculation result is close to the experimental value, which proves its feasibility to small protein–protein systems. Moreover, in order to understand the association mechanism of the insulin dimer, we analyze the variation of SASA, π–π interaction and hydrogen bond interaction during the binding process. And the residues Vla^B12^, Tyr^B16^, Phe^B24^, Phe^B25^, and Tyr^B26^ have the most favorable contributions to the dimerization. The current work may have implications for the development of the insulin analogues.

## Conflicts of interest

There are no conflicts to declare.

## Supplementary Material
